# A Semi-Supervised Transformer-Based Deep Learning Framework for Automated Tooth Segmentation and Identification on Panoramic Radiographs

**DOI:** 10.3390/diagnostics14171948

**Published:** 2024-09-03

**Authors:** Jing Hao, Lun M. Wong, Zhiyi Shan, Qi Yong H. Ai, Xieqi Shi, James Kit Hon Tsoi, Kuo Feng Hung

**Affiliations:** 1 Applied Oral Sciences and Community Dental Care, Faculty of Dentistry, The University of Hong Kong, Hong Kong SAR, China; jinghao@connect.hku.hk (J.H.);; 2 Imaging and Interventional Radiology, Faculty of Medicine, The Chinese University of Hong Kong, Hong Kong SAR, China; lun.m.wong@cuhk.edu.hk; 3 Paediatric Dentistry and Orthodontics, Faculty of Dentistry, The University of Hong Kong, Hong Kong SAR, China; 4 Health Technology and Informatics, The Hong Kong Polytechnic University, Hong Kong SAR, China; 5 Section of Oral Maxillofacial Radiology, Department of Clinical Dentistry, University of Bergen, 5009 Bergen, Norway; xieqi.shi@uib.no

**Keywords:** tooth segmentation, tooth identification, Transformer neural network, semi-supervised learning, deep learning

## Abstract

Automated tooth segmentation and identification on dental radiographs are crucial steps in establishing digital dental workflows. While deep learning networks have been developed for these tasks, their performance has been inferior in partially edentulous individuals. This study proposes a novel semi-supervised Transformer-based framework (SemiTNet), specifically designed to improve tooth segmentation and identification performance on panoramic radiographs, particularly in partially edentulous cases, and establish an open-source dataset to serve as a unified benchmark. A total of 16,317 panoramic radiographs (1589 labeled and 14,728 unlabeled images) were collected from various datasets to create a large-scale dataset (TSI15k). The labeled images were divided into training and test sets at a 7:1 ratio, while the unlabeled images were used for semi-supervised learning. The SemiTNet was developed using a semi-supervised learning method with a label-guided teacher–student knowledge distillation strategy, incorporating a Transformer-based architecture. The performance of SemiTNet was evaluated on the test set using the intersection over union (IoU), Dice coefficient, precision, recall, and F1 score, and compared with five state-of-the-art networks. Paired *t*-tests were performed to compare the evaluation metrics between SemiTNet and the other networks. SemiTNet outperformed other networks, achieving the highest accuracy for tooth segmentation and identification, while requiring minimal model size. SemiTNet’s performance was near-perfect for fully dentate individuals (all metrics over 99.69%) and excellent for partially edentulous individuals (all metrics over 93%). In edentulous cases, SemiTNet obtained statistically significantly higher tooth identification performance than all other networks. The proposed SemiTNet outperformed previous high-complexity, state-of-the-art networks, particularly in partially edentulous cases. The established open-source TSI15k dataset could serve as a unified benchmark for future studies.

## 1. Introduction

Automated tooth segmentation and identification on dental radiographs are essential and fundamental components in establishing digital workflows for diagnosis and treatment planning across various dental specialties [1,2,3]. Accurate tooth segmentation and identification play a crucial role in the subsequent automated localization of dental diseases (e.g., caries, periapical lesions, and periodontal bone loss) and conditions (e.g., dental fillings, restorations, and appliances), as well as treatment planning steps [4]. 

Previous systematic and scoping review articles have documented a range of artificial intelligence (AI) neural networks designed for tooth detection and segmentation on dental panoramic radiographs using supervised deep learning approaches [1,2,3,5]. The majority of these networks were built based on U-Net, a type of convolutional neural network (CNN) [6,7,8,9,10,11,12] (Supplementary Table S1). Zhao et al. developed a CNN that incorporated global and local attention modules for tooth segmentation on panoramic radiographs [6]. Hou et al. proposed a deep learning architecture, Teeth U-Net [7], which enhanced the original U-Net [13] for automated tooth segmentation. Compared to the original U-Net, Teeth U-Net integrated several attention mechanisms, resulting in an improved segmentation performance from Dice metric scores of 92.78% to 94.28%. Wang et al. proposed a multiscale CNN-based network that further enhanced tooth segmentation accuracy on panoramic radiographs [8]. Nagaraju et al. and Lin et al. respectively improved tooth segmentation performance on panoramic radiographs by employing a multi-scale spatial pooling-based panoptic segmentation technique, and a lightweight deep learning method combined with the knowledge consistency training strategy [9,10]. 

Despite their high performance, previous deep learning networks have typically shown lower accuracies in cases involving multiple missing teeth. These cases represent the most challenging group for automatic segmentation, yet are frequently encountered in clinical practices [11,12]. Errors in tooth segmentation and identification for these patients can propagate downstream in the dental workflow, negatively impacting diagnosis and treatment planning steps [12]. Additionally, the diverse appearances of edentulous status on panoramic radiographs necessitate a large volume of training data, which requires impractically labor-intensive and time-consuming manual labeling [2]. Furthermore, previous deep learning networks were built with complex architectures (i.e., multiple subnetworks) incorporating a multitude of parameters while being trained on relatively small datasets. This not only increased the risk of overfitting, causing challenges in applying these models to new, unseen data but also created difficulties in integrating them into existing digital dental workflows. Moreover, these models were evaluated on proprietary, in-house datasets with limited accessibility, adding challenges to performance comparisons among different AI models. 

Efficient training strategies, such as semi-supervised learning, could be a potential solution to this issue [14]. Previous studies have reported the potential benefits of semi-supervised methods in other dental radiology applications [15,16]. Compared to U-Net and its variants, Transformer-based architectures have shown superiority in capturing global dependencies. These architectures allow each position in the feature sequence to attend to all other positions, enabling robust feature extraction across various scales and potentially suitable for tooth segmentation and identification for partially edentulous patients, where recognizing global patterns and relationships is crucial. Nonetheless, it has not been investigated whether Transformer-based architectures developed using a semi-supervised learning method could have the potential to improve automated tooth segmentation and identification on panoramic radiographs, especially for partially edentulous patients. 

Therefore, this study aims to (i) propose a novel semi-supervised Transformer-based framework (SemiTNet) using a label-guided teacher–student knowledge distillation strategy for automated tooth segmentation and identification on panoramic radiographs, (ii) compare the performance of SemiTNet with five state-of-the-art deep learning approaches on the independent test set, for both fully dentate and partially edentulous individuals, as well as for fully dentate individuals and partially edentulous individuals separately, and (iii) establish an open-source dataset (TSI15k) from existing public datasets as a standardized and publicly available benchmark for future studies to compare the performance of different methods on related tasks.

The key contributions of this study can be summarized as follows: (i) The findings demonstrate that the teacher–student knowledge distillation training framework allows better generalizability for tooth segmentation on panoramic radiographs compared to conventional supervised training techniques. This reduces the risk of overfitting without requiring a significant increase in the volume of labeled training data. (ii) Introducing Transformer modules into the proposed SemiTNet contributes to a statistically significant increase in tooth identification accuracy, especially for partially edentulous cases. (iii) The established open-source TSI15k dataset, which includes a total of 16,317 panoramic radiographs from both fully dentate and partially edentulous patients, serves as a unified benchmark for future studies to fairly compare the performance of different novel methods on related tasks.

## 2. Materials and Methods

### 2.1. Training and Testing Datasets (Benchmark TSI15k)

Publicly available dental panoramic radiograph datasets were systematically searched and compiled. Duplicate images from the same institution across different datasets were excluded. Panoramic radiographs with tooth segmentation annotations and teeth labeled using the Federation Dentaire Internationale (FDI) tooth numbering system by experts from previous studies or medical imaging AI challenges were included as the first cohort. Additionally, panoramic radiographs without segmentation annotation and labeling were included as the second cohort. Images from the first cohort were randomly divided into training and test sets at a 7:1 ratio, creating a unified evaluation benchmark with a total of 35,000 boxes and masks. Images from the second cohort were merged into the training set for semi-supervised learning to reduce overfitting to the labeled samples and enhance model robustness. Eventually, a total of 16,317 images were compiled to create the open-source dataset (TSI15k) [16,17,18], consisting of 16,126 training images (1398 labeled and 14,728 unlabeled images) and 191 testing images (Table 1). The TSI15k dataset comprises images with a resolution of approximately 2000 × 1000 pixels, representing individuals with a wide range of dental conditions. This includes patients with fully dentate or partially edentulous, crowded dentition, endodontically treated teeth, dental fillings, and restorations, to name a few.

### 2.2. Network Architecture

**Figure 1:**
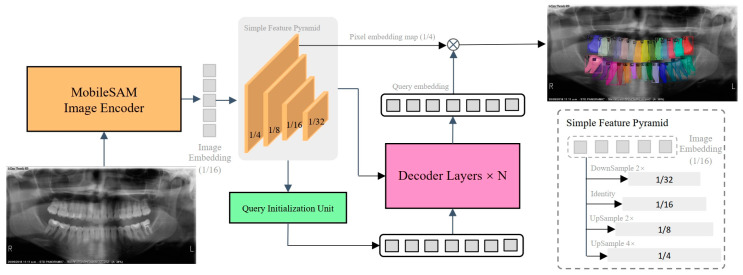
The architecture of the proposed SemiTNet features a streamlined encoder-decoder structure that includes four main components: an image encoder, a basic feature pyramid, a query initialization unit, and a mask decoder. The query initialization unit is responsible for identifying foreground regions within the image, and it uses the associated features to set up the initial queries for the mask decoder.

The network gradient episodic memory (GEM) [19], previously designed and investigated by our team for segmentation tasks in the domain of natural images, was applied for tooth segmentation and identification due to its outstanding performance in feature extraction and object segmentation. The GEM adopts a Transformer-based encoder-decoder architecture consisting of an image encoder, a simple feature pyramid, a query initialization unit, and a mask decoder, as shown in Figure 1. The image encoder was a ViT-Tiny architecture [20] with the MobileSAM pre-trained model [21] that was used to extract the features of panoramic radiographs. To boost the performance for tooth segmentation and identification, the multi-scale feature maps were produced by using the last feature map from the image encoder via a simple feature pyramid following ViTDet [22]. Specifically, the feature maps of scales 1/8, 1/4, and 1/32 were generated using deconvolution of strides 2 and 4 and max-pooling of strides 2, respectively. The feature map of scale 1/4 is directly appointed as the role of the pixel embedding map, which is used to produce the final predictions.

Given these hierarchical feature maps, the query in the decoder was first initialized using a query initialization unit. The hierarchical feature maps were processed by three prediction heads: classification, detection, and segmentation. Each of these heads was identical to their corresponding decoder heads. The hierarchical feature maps underwent a process of aggregation through downsampling and upsampling operations, resulting in the aggregated feature F. Subsequently, feature-wise classification results were derived from F using the Softmax operation, which provided confidence scores for each feature. In order to select the most informative features, all confidence scores were ranked, and the features corresponding to the top-k scores were chosen as the queries.

The classification score of each token was considered the confidence that was used to select the top-ranked features. These selected features were then fed into the decoder as content queries. Additionally, the selected features were used to regress bounding boxes and perform a dot-product with the pixel embedding map to predict masks. Both the predicted boxes and masks were supervised by the ground truth and served as initial anchors for the decoder.

Subsequently, the queries in the decoder were gradually updated through the interaction between the key and the value from hierarchical feature maps as well as the previous query via the cross-attention operation mechanism in each decoder layer. Eventually, the final predictions were obtained by dot-producting [23] each query embedding from the decoder with the pixel embedding map. In summary, a panoramic radiograph I ∈ R^H×W×3^ was fed to the image encoder, and four-scale feature maps C2, C3, C4, and C5 were obtained via a simple feature pyramid P, of which the resolutions were 1/4, 1/8, 1/16, and 1/32 of the input image, respectively. Afterward, the mask decoder took queries Q ∈ R^N×256^ and the flattened three high-level feature maps C3, C4, and C5 as inputs and update queries Q. Nine decoder layers were used in our default experimental settings. Finally, the updated queries Q were dot-multiplied with the pixel embedding map C2 to obtain a predicted mask M. The whole process was formulated as follows:

$$\begin{array}{c} C2,C3,C4,C5=P\left(E\left(I\right)\right),\\ \begin{array}{c} M=C2⊗D(Q,Flatten(C3,C4,C5)), \end{array} \end{array}$$

where ε is the image encoder and D is the mask decoder. The ⊗ indicates the dot production. Note that the prediction masks are output at each decoder layer.

### 2.3. Semi-Supervised Learning

**Figure 2:**
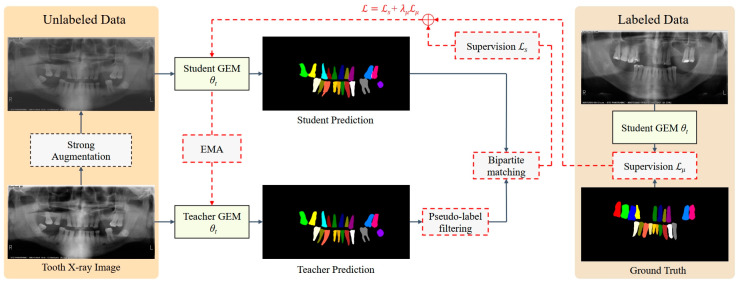
Workflow of the distillation stage in the semi-supervised learning strategy. The original unlabeled panoramic radiographs were fed into the teacher model, while the strongly augmented unlabeled images were fed into the student model. The student model was updated using both the supervised loss (*L_s_*) and unsupervised loss (*L_u_*). The teacher model was subsequently updated using Exponential Moving Average (EMA).

Semi-supervised learning can be used in label-scarce situations by making use of unlabeled data to boost the model’s performance [24,25,26,27]. The label-guided teacher–student knowledge distillation strategy [14] was employed to effectively leverage unlabeled data and enhance the model’s performance, which can be divided into three steps:

(i) Teacher pre-training: The teacher model, parameterized by θt, is exclusively trained on labeled data.
(ii) Enhanced burn-in process: The student model, parameterized by θs, is initialized by the image encoder of MobileSAM [21] and trained on both labeled and unlabeled data using pseudo-labels generated by the teacher model in the first pre-training stage. During this phase, the teacher model remains fixed.
(iii) Distillation stage: In this stage, the student model’s weights are transferred to the teacher model, and continue training the student on both labeled and unlabeled data as before. The teacher model is updated using an exponential moving average (EMA) [28] of the student’s weights. The workflow for this stage is illustrated in Figure 2.

The high-quality pseudo-label was extracted using a straightforward thresholding method that considers both the predicted class probability and the size of the predicted. A predicted mask was selected as a pseudo-label if it meets two criteria: (i) the maximum class probability is above the class threshold pc ≥ αc, and (ii) the size of the predicted mask is above the size threshold ∑PH × W σ($\hat{y}$(p)) ≥ αs where σ represents the sigmoid activation of the binary mask prediction. H and W refer to the height and width of the image, respectively. In our experimental settings, the class threshold αc is 0.7 and the size threshold α_s_ is 5.

### 2.4. Loss Function

During the training phase, the total loss consisted of the supervised ($L_{sup}$) and unsupervised ($L_{unsup}$) losses, which shared the same loss function, defined as follows:

$$\begin{array}{c} L_{total}=L_{sup}+\lambda_{unsup}L_{unsup} \end{array}$$

The unsupervised loss weight *λ_unsup_* was empirically set to 2 in our experiments. The loss function is structured as a weighted sum of five loss components, as follows:

$$\begin{array}{c} L_{sup/unsup}=\lambda_{L1}L_{L1}+\lambda_{giou}L_{giou}+\lambda_{focal}L_{focal\;}+\lambda_{ce}L_{ce\;}+\lambda_{Dice}L_{Dice} \end{array}$$

Specifically, $L_{L1}$ and $L_{Giou}$ [29] were employed for box regression, and their mathematical formulas are defined as follows:

$$\begin{array}{c} L_{L1}=\frac{1}{N}\sum_{i=1}^{N}\left|y_{i}-{\hat{y}}_{i}\right|,\\ \begin{array}{c} L_{\mathrm{GIoU}}=1-(\frac{\left|A\cap B\right|}{\left|A\cup B\right|}-\frac{\left|C-(A\cup B)\right|}{\left|C\right|}), \end{array} \end{array}$$

where *y_i_* denotes the predicted coordinates and ${\hat{y}}_{i}$ refers to the ground truth coordinates. *A* represents the predicted bounding box, and *B* represents the ground truth bounding box. *C* represents the smallest enclosing box that contains both the predicted bounding box *A* and the ground truth bounding box *B*. 

The $L_{focal}$ represents a focal loss [30] designed for classification purposes, and it is defined as follows:

$$\begin{array}{c} L_{\mathrm{focal}}=-\alpha (1-p_{t}{)}^{\gamma }\log(p_{t}), \end{array}$$

where *α* is a balancing factor for class imbalance. *p_t_* is the predicted probability for the ground truth class. *γ* is the focusing parameter that adjusts the rate at easy examples. 

The mask prediction aspect utilizes both cross-entropy loss $L_{ce}$ and Dice loss $L_{Dice}$ [31], and they are defined as follows:

$$\begin{array}{c} L_{ce}=-\sum_{i}{\hat{y}}_{i}\log\left(y_{i}\right),\\ L_{Dice}=1-\frac{2\sum_{i}{\hat{y}}_{i}y_{i}}{\sum_{i}{\hat{y}}_{i}+\sum_{i}y_{i}}. \end{array}$$

### 2.5. Evaluation Metrics

For the tooth segmentation task, the model’s performance was assessed using the widely recognized metrics of intersection over union (IoU) and Dice coefficient, while the tooth identification task was evaluated using the metrics of precision, recall, and F1 score. These five metrics provide a comprehensive representation of the model’s performance.

The mathematical formulas for these five metrics we used to evaluate models are demonstrated as follows:

$$\begin{array}{c} IoU=\frac{\left(Area\;of\;Intersection\right)}{\left(Area\;of\;Union\right)}\\ \mathrm{Dice}\;\mathrm{Coefficient}=\frac{\left(2*Area\;of\;Intersection\right)}{\left(Area\;of\;Prediction+Area\;of\;Truth\right)}\\ \begin{array}{c} Precision=\frac{TP}{TP+FP} \end{array}\\ \begin{array}{c} Recall=\frac{TP}{TP+FN} \end{array}\\ \begin{array}{c} F1\;score=2*\frac{Precision*Recall}{Precision+Recall} \end{array} \end{array}$$

where TP (true positive), FP (false positive), and FN (false negative) follow the conventional definition in a confusion matrix

IoU measures the overlap between the predicted segmentation (prediction) and the ground truth (label). It is calculated as the ratio of the intersection area (the common area between prediction and ground truth) to the union area (the combined area of prediction and ground truth). A higher IoU value indicates better segmentation performance, ranging from 0 to 1 with a value of 1 indicating a perfect overlap. The Dice coefficient is used for evaluating the similarity between predicted segmentation and ground truth. It is calculated as the ratio of twice the intersection area to the sum of the areas of prediction and ground truth. A higher Dice coefficient value indicates better segmentation performance, ranging from 0 to 1 with a value of 1 indicating a perfect match. Precision measures the proportion of correctly identified positive instances out of all instances identified as positive by the model. Recall measures the proportion of correctly identified positive instances out of all actual positive instances. The F1 score, which is the harmonic mean of precision and recall, provides a single metric that balances the trade-off between precision and recall. 

### 2.6. Performance Comparison and Statistical Analysis

The performances of the proposed SemiTNet and five state-of-art deep learning networks (the CNN-based two-stage framework Mask R-CNN [32] and several recently emerged Transformer-based frameworks including MPFormer [33], Mask2Former [34], MaskDINO [35], and GEM [19]) were initially evaluated descriptively for tooth segmentation and identification on the test set independent from the training set. Their performances were assessed for both fully dentate and partially edentulous individuals, as well as separately for the dentate and edentulous groups. Additionally, the differences in the performance evaluation metrics (IoU, Dice, precision, recall, and F1 score) between SemiTNet and other networks were assessed using paired *t*-tests. Furthermore, the number of parameters of different models that could reflect their computational complexity (i.e., a greater number of parameters indicates higher model complexity) were compared. 

### 2.7. Experiment Settings

All experiments were trained on 8 GeForce V100 32G GPUs (NVIDIA Corporation, Santa Clara, CA, USA) for 26,250 iterations with a total batch size of 16. The total training time was 6 h, and the frame per second (FPS) was 0.658. The learning rate was initialized as 1 × 10^−4^, and it was decreased by 0.1 after 24,000 and 25,000 iterations, respectively. The number of queries used in our study was 100. The optimizer AdamW was used to optimize the model parameters. No direction-related data augmentation strategy was used during the training and test stages.

## 3. Results

### 3.1. Overall Performance

**Figure 3:**
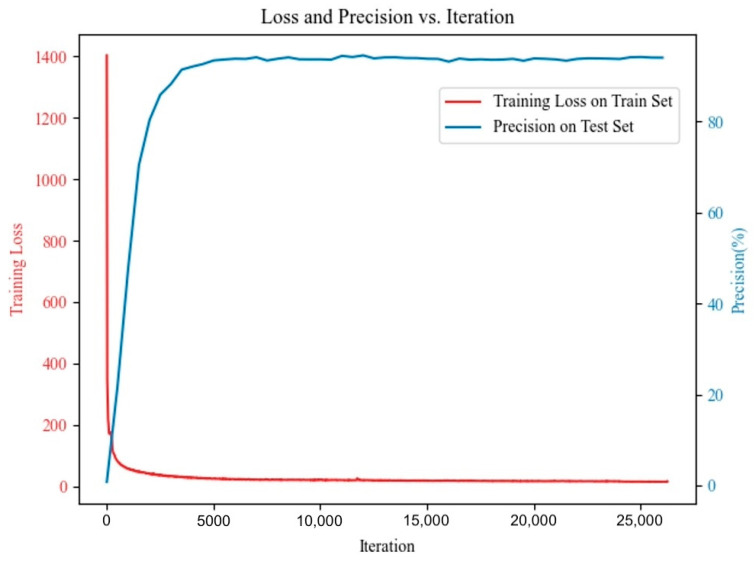
The training loss curve and the precision variation on the test set.

**Figure 4:**
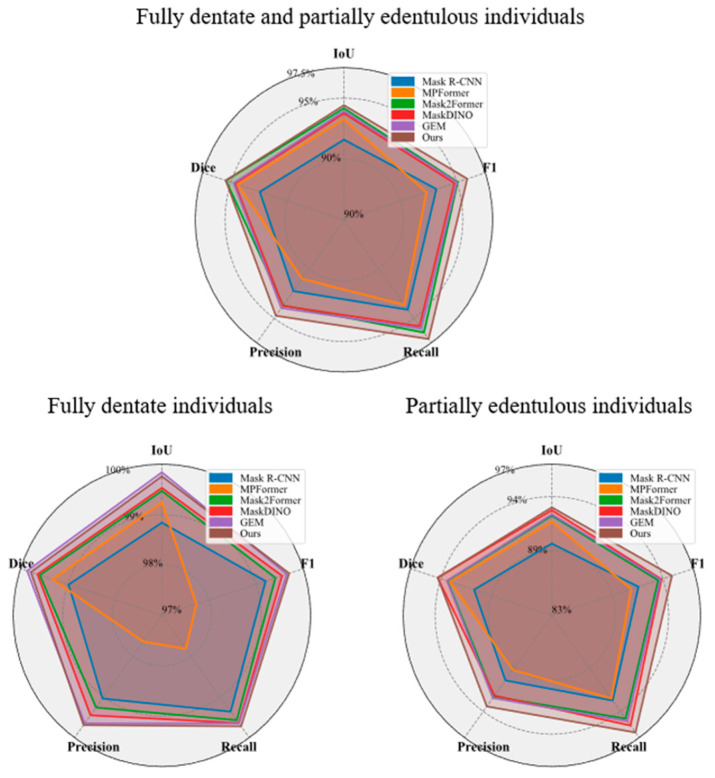
Radar charts visualizing the performance of SemiTNet in comparison to five state-of-art deep learning networks on the test set, for both fully dentate and partially edentulous individuals, as well as for fully dentate individuals and partially edentulous individuals separately.

A total of 191 labeled panoramic radiographs from the independent test set were used to assess the performance of the proposed SemiTNet model and five deep learning networks. The training loss curve and the precision variation on the test set are illustrated in Figure 3. The performance metrics of SemiTNet and other networks on the same test set are detailed in Table 2 and Figure 4. Compared to other networks, the proposed SemiTNet achieved the highest performance for both tooth segmentation (IoU of 94.41% vs. 91.58–94.16% and Dice of 95.45% vs. 92.44–95.43%) and identification (precision of 94.74% vs. 90.99–93.96%, recall of 97.1% vs. 93.63–96.45%, and F1 score of 95.9% vs. 92.29–95.06%) while requiring the minimal model size (number of parameters: 21.6 M vs. 21.6 M–52.0 M). Paired *t*-tests exhibited that SemiTNet achieved statistically significantly higher performance in tooth identification compared to all other networks while its tooth segmentation performance was only statistically significantly higher than that of Mask R-CNN and Mask2Former (Table 2).

### 3.2. Performance Comparison between Fully Dentate and Partially Edentulous Cases

**Figure 5:**
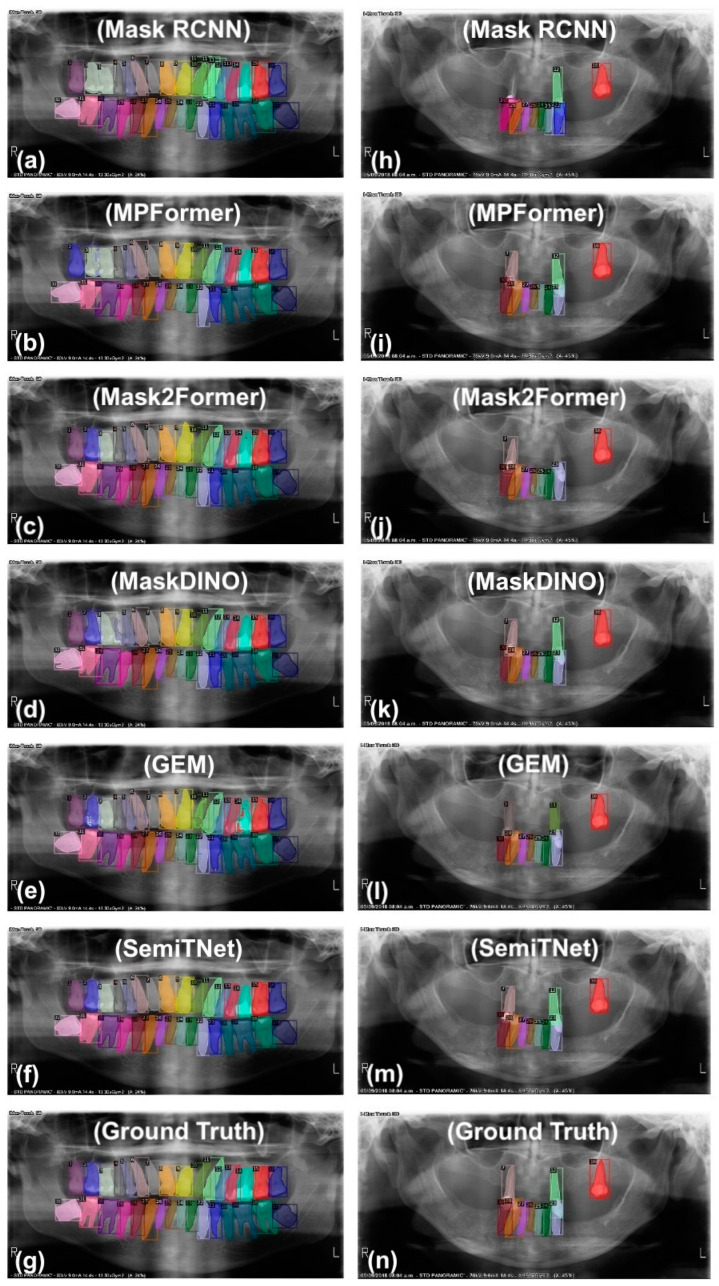
Examples of output generated by SemiTNet and other networks for both fully dentate (**a**–**f**) and partially edentulous (**h**–**m**) cases along with their corresponding ground truth (**g**,**n**).

The performances of SemiTNet and five deep learning networks on the test images from fully dentate individuals (*n* = 40) and partially edentulous (*n* = 151) individuals were investigated, separately. Table 3 and Figure 4 and Figure 5 reveal the differences in the performance of SemiTNet and other networks for fully dentate and/or partially edentulous individuals, respectively. All networks achieved excellent performance in tooth segmentation and identification for fully dentate individuals with all evaluation metrics over 99%. In contrast, performances decrease for partially edentulous individuals (IoU of 89.65–93%, Dice of 90.7–94.31%, precision of 89.23–93.44%, recall of 92.51–96.4%, and F1 score of 90.84–94.89%). 

The proposed SemiTNet achieved near-perfect results for fully dentate individuals with all metrics exceeding 99.69%, and excellent performance for partially edentulous individuals with all metrics over 93%. In the edentulous cases, SemiTNet outperformed the other five networks, achieving an increased IoU of up to 3.35%, Dice of up to 3.60%, precision of up to 4.19%, recall of up to 3.89%, and F1 score of up to 4.05%. Paired *t*-tests confirmed that SemiTNet achieved statistically significantly higher performance in tooth identification for partially edentulous cases compared to all other networks (Table 3).

## 4. Discussion

This study proposed a novel semi-supervised Transformer-based framework (SemiTNet) for automated tooth segmentation and identification on panoramic radiographs and established an open-source dataset (benchmark TSI15k) consisting of 1598 labeled and 14,728 unlabeled images. The SemiTNet not only showed superior performance compared to existing state-of-the-art networks (with an IoU of 94.41%, Dice score of 95.45% for tooth segmentation, and a precision of 94.74%, recall of 97.1%, and an F1 score of 95.9% for tooth numbering) but also significantly reduced the model size, with 21.6 M parameters compared to up to 52 M in other models. In addition, this study’s findings indicate that the teacher–student knowledge distillation training framework allows better generalizability for tooth segmentation on panoramic radiographs compared to conventional supervised training techniques. This reduces the risk of overfitting without necessitating a significant increase in the volume of labeled training data, which is particularly crucial considering the limited scale of dental data (ranging from hundreds to thousands) compared to natural images that can have up to millions of labeled images [1,36]. In this study, SemiTNet was able to exploit a large quantity of unlabeled panoramic radiographs (*n* = 14,728), which is significantly more than the number of training images (ranging from 500 to 1500) used in previous studies [5,6,7,12,37]. This approach alleviates the need for extensive labeling of all included data. 

It has been observed that the previously developed CNN models were more likely to misdetect teeth and misidentify tooth sites for partially edentulous patients on panoramic radiographs, resulting in a high false positive rate of 11.54% [12,37]. This issue could be attributed to both the network structure and the lack of training data. CNNs rely heavily on convolutional operations, which may struggle to capture long-range dependencies across an image. Compared to U-Net, Transformer-based architectures excel in capturing global dependencies by allowing each position in the feature sequence to attend to all other positions, enabling robust feature extraction across various scales. Consequently, Transformer-based architectures are particularly suitable for tasks that require understanding the relationships between distant elements. This capability is especially critical in cases involving multiple missing teeth, where recognizing global patterns and relationships is crucial for the accurate identification of tooth sites. In this study, we introduced Transformer modules, which are especially accustomed to learning inter-patch correlations, into the proposed SemiTNet. SemiTNet not only achieves near-perfect tooth segmentation and identification performance for fully dentate individuals (with over 99.6% across all metrics) but also demonstrates excellent performance for partially edentulous individuals (with all metrics over 93%). In terms of tooth identification performance, the proposed SemiTNet outperformed the other five deep learning networks and achieved statistically significantly higher results in the edentulous cases, achieving an increased IoU of up to 3.35%, Dice of up to 3.6%, precision of up to 4.19%, recall of up to 3.89%, and F1 score of up to 4.05%. Despite SemiTNet’s performance in the edentulous group alone being inferior compared to its performance in fully dentate cases, it is on par with or even higher than the performance of many previously developed deep learning models on a mixed set of fully dentate and partially edentulous patients. 

A key feature of the GEM architecture, which the proposed SemiTNet was built on, is the integration of the segment anything model (SAM) [21]. SAM is a visual foundation model known for its precise segmentation capabilities. This integration eliminates the computationally intensive encoder layer, a fundamental component in Transformer-based architectures. In this study, the backbone and encoder layers in most typical Transformer-based models were replaced with MobileSAM, a specific type of SAM designed for efficient operation on mobile or edge devices while maintaining good performance on semantic segmentation tasks. This replacement reduced the required number of parameters and computational complexity of the model. The results showed that the proposed SemiTNet obtained the highest accuracy for both tooth segmentation and identification tasks with a parameter of 21.6 M, which is considerably lower than the up to 52 M parameters required for other state-of-the-art deep learning networks. The lower complexity of SemiTNet, compared to other networks, makes it a more realistic choice for implementation in systems and integration into digital dental workflows.

There is currently a lack of subjective benchmarks for comparison between the performance of AI models on panoramic radiographs. Specifically, directly comparing the performance of AI models on different test sets with, in particular those with different portions of edentulous patients, can be misleading due to variations in data distribution and quality. To ensure consistent and standardized assessment, this study collected dental panoramic radiographs from various publicly available datasets and established the TSI15k dataset, which includes a total of 16,317 panoramic radiographs from both fully dentate and partially edentulous individuals. The open-source TSI15k dataset can serve as a unified benchmark for future studies to fairly compare the performance of different novel methods on related tasks. 

This study has some limitations. One such limitation is that this study did not evaluate SemiTNet’s performance separately for teeth with specific conditions, such as endodontically treated teeth, residual roots, teeth with fillings, crowns, bridges, or orthodontic appliances. The impact of these dental conditions on the tooth segmentation and identification accuracy of deep learning networks should be investigated in future studies specifically designed for comparative analysis. Additionally, the feasibility and cost-effectiveness of integrating the proposed SemiTNet into a GPU server equipped with a user-friendly interface for real-time image analysis should be further investigated. 

## 5. Conclusions

This study proposed a novel semi-supervised Transformer-based framework designed for automated tooth segmentation and identification on panoramic radiographs. By implementing a semi-supervised learning approach with a label-guided teacher–student knowledge distillation strategy and incorporating the GEM architecture, SemiTNet achieved excellent performance in tooth segmentation and identification for both fully dentate and partially edentulous individuals. It outperformed previously proposed high-complexity, state-of-the-art deep learning networks, particularly in partially edentulous cases. The established open-source TSI15k dataset could serve as a unified benchmark for future studies. The code and dataset are available, respectively, at https://github.com/isbrycee/SemiTNet (accessed on 4 August 2024) and https://huggingface.co/datasets/Bryceee/TISI15k-Dataset/blob/main/TISI15k-Dataset.tar (accessed on 4 August 2024).

## Figures and Tables

**Table 1:** The distribution of training and test images in the TSI15k dataset.

		TSI15k Dataset	
		Training Set	Test Set
Cohort 1	Labeled images	1398	191
Cohort 2	Unlabeled images	14,728	0

**Table 2:** Performance comparisons of SemiTNet and five deep learning networks on the test set.

Networks	Segmentation		Identification			Parameters (M)
	IoU (%)	Dice (%)	Precision (%)	Recall (%)	F1 Score (%)	
Mask R-CNN	91.58 * (*p* < 0.001)	92.44 * (*p* < 0.001)	92.24 * (*p* < 0.001)	94.13 * (*p* < 0.001)	93.17 * (*p* < 0.001)	44.5
MPFormer	93.26 * (*p* = 0.002)	94.39 * (*p* = 0.006)	90.99 * (*p* < 0.001)	93.63 * (*p* < 0.001)	92.29 * (*p* < 0.001)	43.9
Mask2Former	94.16	95.43	93.70 * (*p* = 0.002)	96.45	95.06 * (*p* = 0.014)	44.0
MaskDINO	93.75	94.64	93.74 * (*p* = 0.010)	95.81 * (*p* = 0.050)	94.76 * (*p* = 0.022)	52.0
GEM	93.92	94.75 * (*p* = 0.043)	93.96 * (*p* = 0.013)	96.04 * (*p* = 0.005)	94.99 * (*p* = 0.006)	21.6
SemiTNet (ours)	94.41	95.45	94.74	97.10	95.90	21.6

IoU, intersection over union; M, million. Paired *t*-tests were performed to compare the evaluation metrics between SemiTNet and other networks, with *p* values and asterisk shown only if significant (≤0.05). Paired *t*-tests exhibited that SemiTNet achieved statistically significantly higher performance in tooth identification compared to all other networks while its tooth segmentation performance was only statistically significantly higher than that of Mask R-CNN and Mask2Former.

**Table 3:** Comparison of SemiTNet with five deep learning networks for fully dentate and partially edentulous cases.

	Fully Dentate Individuals (*n* = 40)					Partially Edentulous Individuals (*n* = 151)				
Network	Segmentation		Identification			Segmentation		Identification		
	IoU (%)	Dice (%)	Precision (%)	Recall (%)	F1 Score (%)	IoU (%)	Dice (%)	Precision (%)	Recall (%)	F1 Score (%)
MaskR-CNN	98.84 * (*p* = 0.033)	98.98 * (*p* = 0.040)	99.04	99.36	99.20	89.65 * (*p* < 0.001)	90.70 * (*p* < 0.001)	90.44 * (*p* < 0.001)	92.74 * (*p* < 0.001)	91.57 * (*p* < 0.001)
MPFormer	99.24	99.32	97.64 * (*p* = 0.004)	97.82 * (*p* = 0.002)	97.73 * (*p* = 0.003)	91.67 * (*p* = 0.004)	93.09 * (*p* = 0.011)	89.23 * (*p* < 0.001)	92.51 * (*p* < 0.001)	90.84 * (*p* < 0.001)
Mask2Former	99.47	99.59	99.26	99.57	99.41	92.24	93.33	92.28 * (*p* = 0.003)	94.82	93.53 * (*p* = 0.018)
MaskDINO	99.53	99.64	99.45	99.64	99.55	92.74	94.31	92.18 * (*p* = 0.017)	95.61	93.86 * (*p* = 0.034)
GEM	99.84	99.86	99.65	99.65	99.65	92.35	93.39 * (*p* = 0.038)	92.46 * (*p* = 0.013)	95.09 * (*p* = 0.006)	93.76 * (*p* = 0.006)
SemiTNet (ours)	99.76	99.78	99.69	99.72	99.70	93.00	94.30	93.42	96.40	94.89

IoU, intersection over union. Paired *t*-tests were performed to compare the evaluation metrics between SemiTNet and other networks, with *p* values and asterisk shown only if significant (≤0.05). Paired *t*-tests exhibited that SemiTNet obtained statistically significantly higher performance in tooth identification for partially edentulous cases compared to all other networks.

## Data Availability

The TSI15k dataset can be accessed at https://huggingface.co/datasets/Bryceee/TISI15k-Dataset/blob/main/TISI15k-Dataset.tar (accessed on 4 August 2024). The code used in this study is available at https://github.com/isbrycee/SemiTNet (accessed on 4 August 2024).

## Supplementary Materials

The following supporting information can be downloaded at https://www.mdpi.com/article/10.3390/diagnostics14171948/s1, Table S1. Previous deep learning networks were proposed for tooth segmentation and identification on panoramic radiographs.
