# Supplementary material for: A Semi-Supervised Transformer-Based Deep Learning Framework for Automated Tooth Segmentation and Identification on Panoramic Radiographs
Source: Diagnostics (Basel). 2024 Sep 3;14(17):1948. doi: 10.3390/diagnostics14171948 (PMC11394203; doi:10.3390/diagnostics14171948)
Supplement: Supplementary file 1 [file diagnostics-14-01948-s001.zip › diagnostics-3168243-supplementary.pdf]

Supplementary Materials:

Supplementary Table S1. Previous deep learning networks proposed for tooth segmentation and identification on panoramic radiographs

Study	Year	Model Architecture	Dataset	Performance
Zhao et al. [6]	2020	CNN with global and local attention modules	1500	Dice=92.72%<br>Recall=93.77%
Hou et al. [7]	2023	Multitask learning architecture including graph convolution network and two CNNs	1500	Precision=95.62%<br>Recall=94.51%<br>Dice=94.28%
Wang et al. [8]	2024	CNN with squeeze-excitation module, a dense skip connection, multi-scale aggregation attention Block, and dilated hybrid self-attentive block	2116	Dice=96.29%<br>F1 score=98.49%
Nagaraju [9]	2024	CNN with multi-scale spatial pooling method	Not clear	IoU=87%<br>F1 score=98.9%<br>Recall=93%<br>Precision=94.5%<br>dice score of 94.5%
Lin et al. [10]	2023	CNN with nonlinear relationship between the spatial attention feature maps of teacher and student networks	1500	Dice= 89%,
Chandrashekar et al. [11]	2024	Two CNN models for incorporating tooth segmentation and identification created independently	1500	F1 score=98.83%
Putra et al. [12]	2024	CNN with one-stage object detection algorithm	500	Precision=88.5%<br>Recall=87.70%
